# Supplementary material for: FastqPuri: high-performance preprocessing of RNA-seq data
Source: BMC Bioinformatics. 2019 May 3;20:226. doi: 10.1186/s12859-019-2799-0 (PMC6500068; doi:10.1186/s12859-019-2799-0)
Supplement: Supplementary file 2 — Archive of FastqPuri. Archive containing all files needed to install and run FastqPuri v1.0.6. Date stamp March 22, 2019. (GZ 47,819 kb) [file 12859_2019_2799_MOESM2_ESM.gz › FastqPuri-1.0.6/html/trimDS_8h_source.html]

FastqPuri: include/trimDS.h Source File


FastqPuri


- include

trimDS.h

Go to the documentation of this file.

1 /\*\*\*\*\*\*\*\*\*\*\*\*\*\*\*\*\*\*\*\*\*\*\*\*\*\*\*\*\*\*\*\*\*\*\*\*\*\*\*\*\*\*\*\*\*\*\*\*\*\*\*\*\*\*\*\*\*\*\*\*\*\*\*\*\*\*\*\*\*\*\*\*\*\*\*\*

2  \* Copyright (C) 2017 by Paula Perez Rubio \*

3  \* \*

4  \* This file is part of FastqPuri. \*

5  \* \*

6  \* FastqPuri is free software: you can redistribute it and/or modify \*

7  \* it under the terms of the GNU General Public License as \*

8  \* published by the Free Software Foundation, either version 3 of the \*

9  \* License, or (at your option) any later version. \*

10  \* \*

11  \* FastqPuri is distributed in the hope that it will be useful, \*

12  \* but WITHOUT ANY WARRANTY; without even the implied warranty of \*

13  \* MERCHANTABILITY or FITNESS FOR A PARTICULAR PURPOSE. See the \*

14  \* GNU General Public License for more details. \*

15  \* \*

16  \* You should have received a copy of the GNU General Public License \*

17  \* along with FastqPuri. \*

18  \* If not, see <http://www.gnu.org/licenses/>. \*

19  \*\*\*\*\*\*\*\*\*\*\*\*\*\*\*\*\*\*\*\*\*\*\*\*\*\*\*\*\*\*\*\*\*\*\*\*\*\*\*\*\*\*\*\*\*\*\*\*\*\*\*\*\*\*\*\*\*\*\*\*\*\*\*\*\*\*\*\*\*\*\*\*\*\*\*\*/

20

29 #ifndef \_DSTRIM\_H

30 #define \_DSTRIM\_H

31

32 #include "fq\_read.h"

33 #include "defines.h"

34

38 typedef struct \_ds\_adap {

39  char ad1[READ\_MAXLEN];

40  char ad2[READ\_MAXLEN];

41  int L1;

42  int L2;

43 } DS\_adap;

44

45 DS\_adap init\_DSadap(char \*ad1, char \*ad2, int L1, int L2);

46

47 int trim\_adapterDS(DS\_adap \*ptr\_DSad, Fq\_read \*r1, Fq\_read \*r2);

48

56 #endif // endif \_DSTRIM\_H

init\_DSadap

DS\_adap init\_DSadap(char \*ad1, char \*ad2, int L1, int L2)

initialization of a DS\_adap structure

**Definition:** trimDS.c:49

\_ds\_adap::L2

int L2

**Definition:** trimDS.h:42

\_fq\_read

stores a fastq entry

**Definition:** fq\_read.h:37

defines.h

Macro definitions.

trim\_adapterDS

int trim\_adapterDS(DS\_adap \*ptr\_DSad, Fq\_read \*r1, Fq\_read \*r2)

trim the sequences, discard them or keep them unchanged depending on them having adapters remnants...

**Definition:** trimDS.c:234

\_ds\_adap

structure containing an adapter pair (for read 1 and read 2)

**Definition:** trimDS.h:38

fq\_read.h

fastq entries manipulations (read/write)

\_ds\_adap::L1

int L1

**Definition:** trimDS.h:41

DS\_adap

struct \_ds\_adap DS\_adap

structure containing an adapter pair (for read 1 and read 2)

\_ds\_adap::ad1

char ad1[READ\_MAXLEN]

**Definition:** trimDS.h:39

\_ds\_adap::ad2

char ad2[READ\_MAXLEN]

**Definition:** trimDS.h:40


---

Generated on Mon Mar 19 2018 23:42:01 for FastqPuri by  

 1.8.14
